# Relationship between human serum albumin and in-hospital mortality in critical care patients with chronic obstructive pulmonary disease

**DOI:** 10.3389/fmed.2023.1109910

**Published:** 2023-04-27

**Authors:** Ma Ling, Li Huiyin, Chen Shanglin, Li Haiming, Di Zhanyi, Wang Shuchun, Bai Meng, Lu Murong

**Affiliations:** ^1^Department of Respiratory, Zhumadian Central Hospital, Zhumadian, China; ^2^Department of Critical Care, The Second Affiliated Hospital of Guangzhou Medical University, Guangzhou, China; ^3^Department of Laboratory, The Fifth People’s Hospital of Panyu District, Guangzhou, China; ^4^Department of Computer Center, Fujian Provincial Maternity Hospital, Fuzhou, Fujian, China

**Keywords:** severe chronic obstructive pulmonary disease, serum albumin, in-hospital mortality, ICU - intensive care unit, relationship

## Abstract

**Background:**

The relationship between human serum albumin levels and the prognosis of critical care patients with chronic obstructive pulmonary disease (COPD) remains controversial.

**Objective:**

To investigate the relationship between serum albumin levels and in-hospital mortality in critical care patients with COPD. METHODS: This study used a retrospective observational cohort from the Medical Information in Intensive Care database (MIMIC-IV) in the United States. Multivariate Cox regression analysis was used to assess the relationship between serum albumin levels and in-hospital mortality. A restricted cubic spline line was also used to explore nonlinear relationship.

**Results:**

A total of 3,398 critical care patients with COPD were included. The overall in-hospital mortality was 12.4%. We found a negative relationship between human serum albumin and in-hospital mortality (HR = 0.97, 95% CI 0.96–0.99, *p* = 0.002).

**Conclusion:**

In critical care patients with COPD, there was a negative association between human serum albumin and in-hospital mortality.

## 1. Introduction

Chronic obstructive pulmonary disease (COPD) is currently a major public health problem worldwide, with a global prevalence of 11.7% in 2010 (1). COPD is also the third leading cause of death worldwide (2). Patients with acute exacerbation of COPD often require hospitalization, or even need intensive care unit admission (2). Identifying the reversible risk factors during hospitalization is therefore essential to help reduce mortality as well as the burden of disease.

Human serum albumin is a multifunctional plasma protein that accounts for more than 50% of the total plasma protein content. Physiologically, human serum albumin, in addition to its antioxidant properties (3), is also an acute-phase response protein whose concentration decreases during the acute-phase response (4). Albumin is also a clinical bio-marker of malnutrition. Hypoproteinemia is associated with prolonged hospital stay during acute exacerbations, acute respiratory failure and increased mortality in COPD patients (3, 5, 6). However, other studies reported no correlation between albumin level and prognosis in COPD patients (7).

In this study, we attempted to identify the dose–response relationship between human serum albumin level and hospital mortality in COPD patients using a large data set of critical illnesses (8).

## 2. Data and methods

### 2.1. Methods

This study used a retrospective observational cohort of all patients diagnosed with COPD in the Medical Information in Intensive Care database (8) (MIMIC-IV version 1.0). MIMIC-IV is a large, real-world clinical database of critical care patients from 2008 to 2019 in Beth Israel Deaconess Medical Center (9). One of the authors (Shanglin Chen) was granted access to utilize the database (license number: 10756765). This study was written according to the Strengthening the Reporting of Observational Studies in Epidemiology guidelines (10). All the data accessed complied with relevant data protection and privacy regulations.

### 2.2. Study population

This study was based on the real-world study concept and included all COPD patients from the MIMIC-IV database. The inclusion criteria: 1. First admission to the intensive care unit. 2. Human serum albumin level measured within 24 h. 3. Age ≥ 18 years.

### 2.3. Exposure and covariates

This study focused on assessing the relationship between baseline human serum albumin and hospital mortality in patients with severe COPD. Patients were divided into hypoalbuminemia group (<30 g/L) and hyperalbuminemia group (≥30 g/l) according to previous studies (11, 12). Vital signs and laboratory indicators were analyzed using the worst value within 24 h of ICU admission. Covariates were identified based on previous serum albumin-related studies and clinical relevance (10–13). Covariates in this study including age, sex, Glasgow score, mean arterial pressure, respiratory rate, oxygen saturation, serum sodium, serum potassium, blood creatinine, hemoglobin, platelets, myocardial infarction, heart failure, peripheral vascular disease, cerebrovascular disease, renal disease, cancer, diabetes mellitus, co-morbidity index, SAPSII score, SOFA score, and OASIS score.

### 2.4. Outcome

The outcome was in-hospital mortality.

### 2.5. Statistical analysis

Data analysis was performed using R.3.3.2, free statistical software version 1.6.1 (13). Categorical variables were expressed as percentages (%) using the *χ*2 test; continuous variables with normal distribution were expressed as mean ± standard deviation (x ± s) and t-test were used for comparison between groups. Continuous variables with skewed distribution were expressed as median (quartiles) using Kruskal-Wallis test. Missing data were replaced by dummy variables (14). Multivariable Cox regression analysis was used to analyze the independent association between serum albumin level and in-hospital mortality. A restricted cubic spline function was used to analyze the nonlinear relationship between human serum albumin and hospital mortality, and a log-likelihood ratio test of *p* < 0.05 was used to consider the relationship as nonlinear. Kaplan–Meier survival curves for low and high human serum albumin were plotted and Multivariable Cox regression analysis was performed. *p* < 0.05 (two-sided) was considered a statistically significant difference. Propensity-score matching (PSM) was also used to compare the outcomes between two groups.

### 2.6. Missing data

There are many variables with missing data in the MIMIC IV database. For PaO_2_/FIO_2_ ratio, over 20% of the values were missing and were removed from this analysis. In other continuous variables, the missing values were imputed by k-Nearest Neighbors method (15).

## 3. Results

### 3.1. Study population

3,398 COPD patients were screened in the MIMIC-IV database who met the inclusion and exclusion criteria of this study.

### 3.2. Baseline characteristics

Of 3,398 COPD patients, age was 70.3 ± 12.0 years. 1857 (54.6%) were male. The overall in-hospital mortality was 12.4%. 1,062 patients had low human serum albumin (<30 g/L) and 2,336 patients had human serum albumin ≥30 g/L. Glasgow score, mean arterial pressure, respiratory rate, serum potassium, hemoglobin, platelets, PO_2_, PCO_2_, heart failure, cancer, diabetes mellitus, sepsis, MV, AECOPD, co-morbidity index, and co-morbidity index SAPSII score, SOFA score, and OASIS score were imbalance in two groups (all *p* < 0.05) disease.

### 3.3. Association between baseline human serum albumin and in-hospital mortality

#### 3.3.1. Univariate and multivariate cox regression analyses

**Figure 1:**
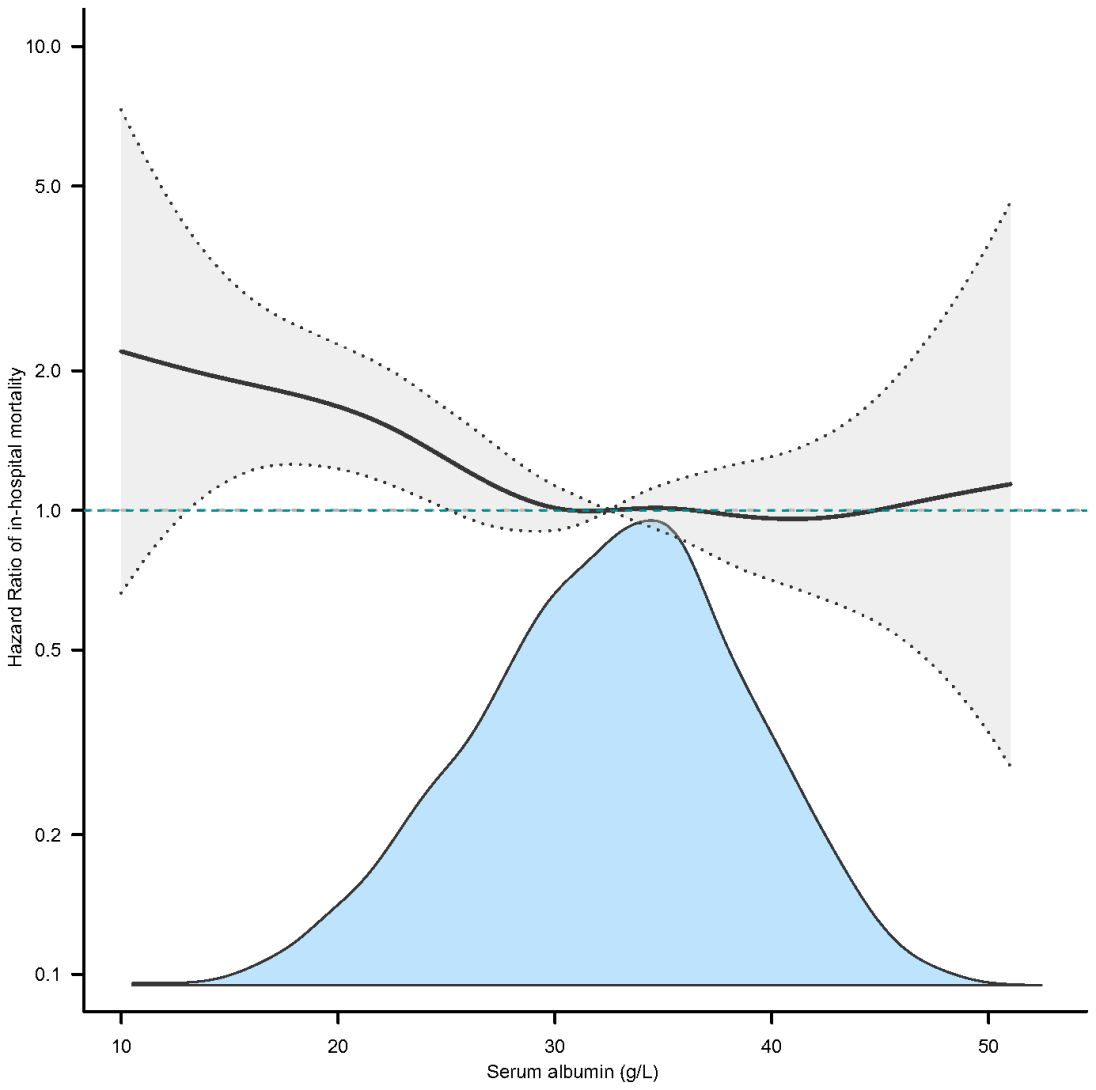
Nonlinear relationship between serum albumin and in-hospital mortality (adjusted all covariates in Table 1).

In the extended multivariable Cox models, we observed that the hazard ratios (HRs) of serum albumin were consistently significant in unadjusted, minimally adjusted (adjusted for age and sex), and fully adjusted (adjusted for all covariates in Tables 1, 2; Supplementary Table S1). The fully adjusted model indicated a negative association between serum albumin level and in-hospital mortality (HR = 0.97, 95% CI 0.96–0.99, *p* = 0.002. Restricted cubic spline analysis (Figure 1) showed nonlinear relationship between human serum albumin and in-hospital mortality (nonlinear test: *p* = 0.028, Table 3). When human serum albumin was <30 g/L, each 1 g/L increase in human serum albumin was associated with a 5% reduction in hospital mortality (HR = 0.95, 95% CI (0.91, 0.98), *p* = 0.002). While human serum albumin was ≥30 g/L, increased serum albumin was not associated with changing in hospital mortality (HR = 1.00, 95% CI 0.96–1.04, *p* = 0.775) (Table 3).

**Table 1:** Baseline characteristics of participants.

	All patients	Albumin<30 g/L	Albumin≥30 g/L	
Covariates	(*n* = 3,398)	(*n* = 1,062)	(*n* = 2,336)	*p* value
Age(years)	70.3 ± 12.0	70.3 ± 11.6	70.3 ± 12.2	0.474
Sex, n (%)(male)	1857 (54.6)	583 (54.9)	1,274 (54.5)	0.645
Glasgow score	14.0 (12.0, 15.0)	14.0 (11.0, 15.0)	14.0 (13.0, 15.0)	< 0.001
MAP (mmHg)	77.9 ± 10.9	74.6 ± 9.5	79.4 ± 11.2	< 0.001
Respiratory rate (BPM)	29.3 ± 6.6	29.9 ± 6.6	29.1 ± 6.6	< 0.001
Oxygen saturation(%)	96.0 ± 2.7	96.1 ± 3.6	96.0 ± 2.2	0.258
Serum sodium(mmol/L)	136.2 ± 5.8	136.0 ± 6.0	136.4 ± 5.6	0.078
Serum potassium(mmol/L)	4.8 ± 1.0	4.8 ± 1.0	4.9 ± 1.1	0.039
Creatinine (mg/L)	1.3 (0.9, 2.1)	1.3 (0.9, 2.3)	1.2 (0.9, 2.1)	0.208
Hemoglobin (g/L)	9.8 ± 2.3	8.8 ± 2.0	10.2 ± 2.3	< 0.001
Platelets(10^9^/L)	182.0 (126.0, 244.0)	165.0 (100.0, 239.0)	189.0 (137.0, 245.8)	< 0.001
PO_2_	97.0 (59.8, 176.0)	91.0 (58.0, 165.2)	109.0 (66.8, 198.0)	< 0.001
PCO_2_	52.0 (43.0, 65.0)	52.5 (43.0, 66.0)	50.5 (42.0, 61.0)	< 0.001
Myocardial infarction	748 (22.0)	226 (21.3)	522 (22.3)	0.487
Heart failure	1,662 (48.9)	459 (43.2)	1,203 (51.5)	< 0.001
Peripheral vascular disease	570 (16.8)	170 (16)	400 (17.1)	0.420
Cerebrovascular disease	411 (12.1)	120 (11.3)	291 (12.5)	0.337
Renal disease	1,109 (32.6)	324 (30.5)	785 (33.6)	0.074
Cancer	504 (14.8)	224 (21.1)	280 (12)	< 0.001
Diabetes mellitus	1,305 (38.4)	382 (36)	923 (39.5)	0.049
Sepsis	2,151 (63.3)	818 (77)	1,333 (57.1)	< 0.001
MV				< 0.001
Noninvasive-Ventilation	235 (6.9)	53 (5.0)	182 (7.8)	
Invasive-Ventilation	875 (25.8)	377 (35.5)	498 (21.3)	
No-Ventilation	2,288 (67.3)	632 (59.5)	1,656 (70.9)	
Co-morbidity index	7.4 ± 2.7	7.8 ± 2.8	7.3 ± 2.6	< 0.001
SAPSII score	39.0 ± 13.2	43.7 ± 13.8	36.9 ± 12.4	< 0.001
SOFA score	5.8 ± 3.8	7.4 ± 4.2	5.1 ± 3.4	< 0.001
OASIS score	33.5 ± 9.1	36.7 ± 9.4	32.1 ± 8.6	< 0.001
AECOPD	1,268 (37.3)	376 (35.4)	892 (38.2)	0.012

MAP, mean arterial pressure; SAPS II score, Simplified Acute Physiology Score II; SOFA score, Sequential Organ Failure Score; OASIS score, Oxford Acute Severity of Illness Score; PO_2_, Partial pressure of oxygen; PCO_2_, Partial pressure of carbon dioxide; MV, Mechanical ventilation; COPD, chronic obstructive pulmonary.

**Table 2:** Relationship between serum albumin and in-hospital mortality.

Exposure	Unadjusted model HR(95% CI)	P value	Minimally adjusted model HR(95% CI)	*p* value	Fully adjusted model HR(95% CI)	*p* value
Serum albumin	0.94 (0.93, 0.96)	<0.001	0.94 (0.92, 0.95)	<0.001	0.97 (0.96, 0.99)	0.002
Grouped serum albumin						
<30 g/L	Reference		Reference		Reference	
≥30 g/L	0.51 (0.42, 0.62)	<0.001	0.49 (0.41, 0.60)	<0.001	0.75 (0.60, 0.93)	0.008

Unadjusted model: no adjustment for covariates.

Minimally adjusted model: adjusted for age and gender.

Fully adjusted model: adjusted all covariates in Table 1.

**Table 3:** Nonlinear relationship between serum albumin and in-hospital mortality.

Serum albumin	HR (95% CI)	*P* value
<30 g/L	0.95 (0.91, 0.98)	0.002
≥30 g/L	1.00 (0.96, 1.04)	0.775
Nonlinear test		0.028

Adjusted all covariates in Table 1.

#### 3.3.2. The in-hospital mortality was 21.7% (230/1062) and 8.2% (192/2336) for low and hyperalbuminemia groups, respectively

**Figure 2:**
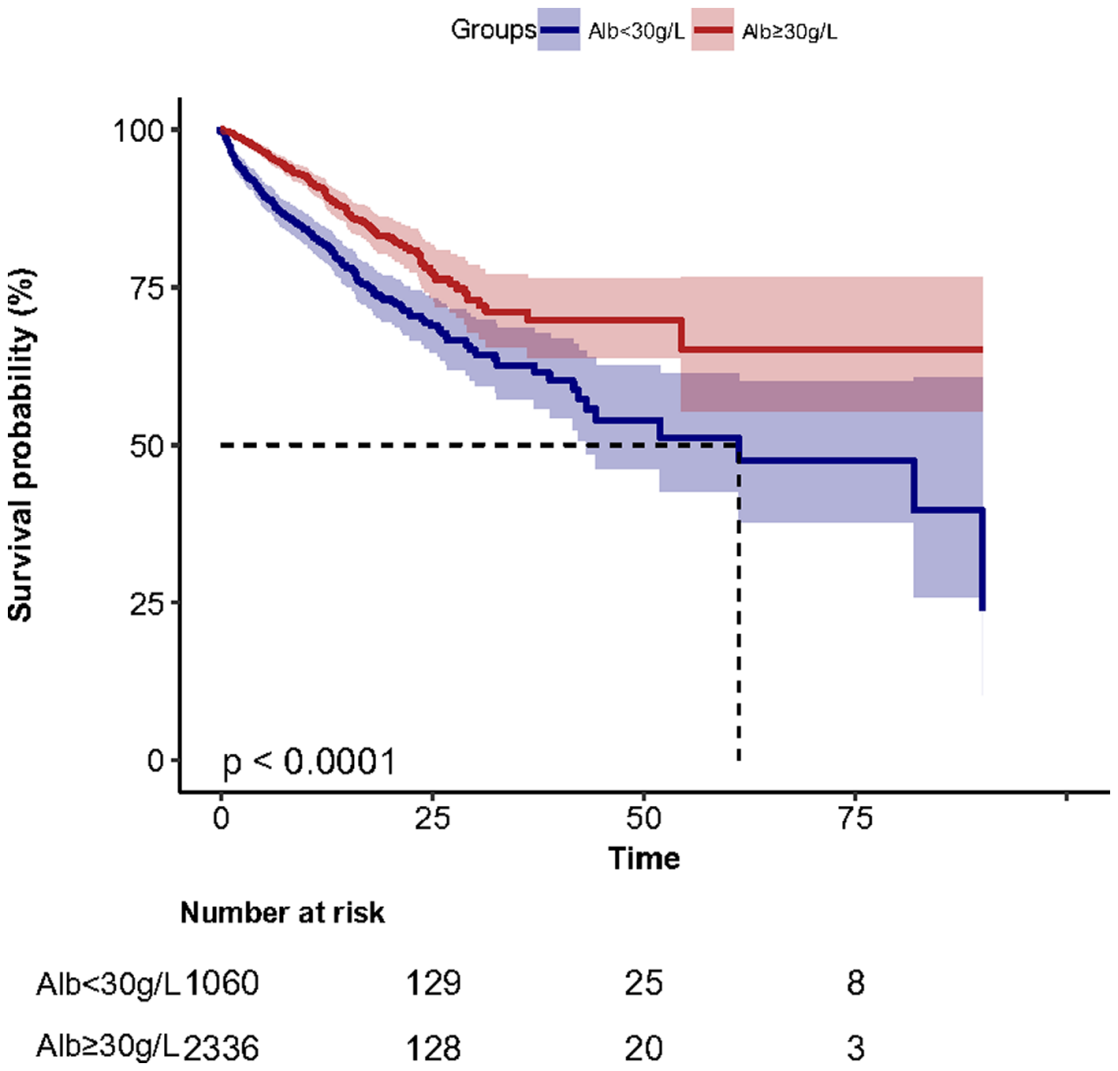
Survival curves in the low albumin and high albumin groups.

Kaplan–Meier survival curves (Figure 2) for cumulative hospital survival showed a statistically significant difference between the low and high human serum albumin groups (Log-rank test: *p* < 0.001). Multivariable Cox regression analysis showed that after adjusting for all confounders in Table 1, the in-hospital mortality in high human serum albumin group was 0.25 times lower than in the low human serum albumin group (HR = 0.75, 95% CI: 0.60 to 0.93, *p* = 0.008, Table 2). In Multivariable Cox regression analysis, age, Glasgow score, respiratory rate, oxygen saturation, serum potassium, PCO_2_, peripheral vascular disease, renal disease, diabetes mellitus, co-morbidity index, SOFA score were also associated with in-hospital mortality (Supplementary Table S1).

**Figure 3:**
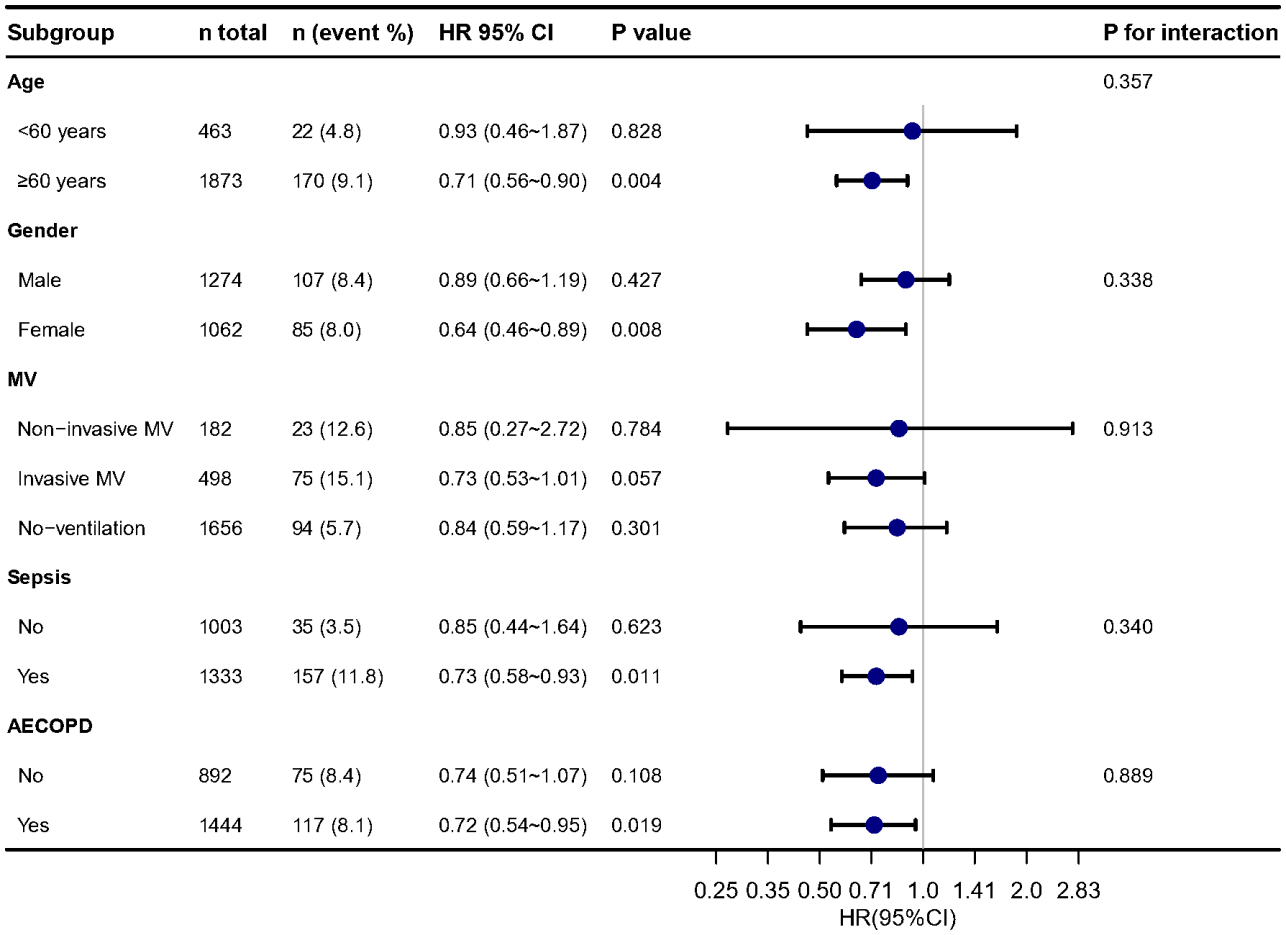
Stratified analysis of relationship between serum albumin and in-hospital mortality. Adjusted for all cofounders in Table 1.

The distribution of albumin levels in those that survived versus those non-survived was 30.0 ± 6.2 and 28.9 ± 7.0 g/L (Supplementary Figure S3). In subgroup analysis (Figure 3), we found the results were stable in different age, sex, MV, sepsis and AECOPD groups. In multivariable Cox regression models, we evaluating the association between serum albumin and 30-day, 90-day and 1-year mortality, the results remained stable.

## 4. Discussion

The results of this study showed that in ICU, lower human serum albumin is negatively associated with higher in-hospital mortality in patients with COPD. Analysis of dose–response relationship using restricted cubic spline functions indicate a nonlinear relationship between human serum albumin and in-hospital mortality. When human serum albumin was below 30 g/L, there was a negative correlation between human serum albumin and in-hospital mortality. When human serum albumin is above or equal to 30 g/L, the correlation was not significant.

As an important biomarker of nutritional status, lower serum albumin was reportedly associated with worsening clinical status during COPD (16, 17). We also found the same effect in COPD patients with critical illness. Previous studies had also reported that low levels of serum albumin could increase in-hospital mortality in COPD patients. In a retrospective (*n* = 574) cohort study in Spain, serum albumin levels were found to be strongly associated with disease severity and outcome in elderly patients with COPD (6). In another smaller study (*n* = 20), albumin levels were also indicated to be closely related to the prognosis of COPD patients (18). Similar findings were found in a larger (*n* = 1,647) cohort (19). However, compared to these studies, the present cohort study had a larger sample size (*n* = 3,398), adjusted for more confounding factors, and had relatively more stable and reliable results.

It has also been shown that there was no relationship between hypoalbuminemia and patient prognosis in a cohort study (7). However, as a result of the small sample size of this study (*n* = 431) and the large difference in the distribution of the number of people in the low and high human serum albumin groups in the cohort (1.5% versus 98.5%), the results were not consisting with our study (HR of low albumin versus normal was 1.67 (0.22, 12.57), *p* = 0.604).

This study also has clinical implications. We found a nonlinear relationship between human serum albumin and hospital mortality in critical care patients with COPD. Our clinical experience also supports this nonlinear relationship. The optimal cut off point for in-hospital mortality was 30 g/L which was similar with previous study (11, 12). Ruiqi Chen et al. found that an albumin level of 30.5 g/l was best to predict the prognosis for in-hospital mortality (11). This cut off point also suggests that clinicians may consider using albumin to increase albumin levels in patients with COPD when human serum albumin is low.

Albumin is a biomarker of malnutrition and frailty in older patients (20). Therefore, we did subgroup analysis based on age groups (>60 years and < 60 years). It seems no different between the two groups. However, for those in the >60 years group higher albumin is associated with lower mortality, while this is not seen in the <60 years group. We looked at the relationship between age and albumin and found that younger patients tended to have higher albumin levels (Supplementary Figure S1A). These patients would fall more on the right side of the curve fit, i.e., after albumin levels above 30 g/L. At this region, the increased serum albumin was not associated with changing in hospital mortality (Supplementary Figure S1B).

Similar to the previous study and our clinical experience, we also found age (21, 22), Glasgow score (23), respiratory rate (24), oxygen saturation (25), serum potassium, PCO_2_, peripheral vascular disease, renal disease, diabetes mellitus, comorbidity index (26), SOFA score (27) were also associated with in-hospital mortality.

## 5. Limitations

There are some limitations of this study. First, this is a retrospective observational single-center study and the effect of uncontrolled confounding factors cannot be excluded, however, the large sample size of this study may reduce bias to some extent. Secondly, this study evaluated the most unwell patients (in the ICU setting) and the findings cannot be applied to hospitalized patients who do not end up needing ICU level care. Third, In the study, data on respiratory-related death could not be found in MIMIC-IV database, further studies need to clarify whether albumin is associated with respiratory-related death in COPD patients.

## 6. Conclusion

In critical care patients with COPD, there was a negatively association between human serum albumin and in-hospital mortality.

## Data availability statement

The original contributions presented in the study are included in the article/Supplementary material, further inquiries can be directed to the corresponding authors.

## Ethics statement

Written informed consent was obtained from the individual(s) for the publication of any potentially identifiable images or data included in this article.

## Author contributions

ML and LHu wrote the manuscript. BM and CS conducted the data analysis. LHa modified the manuscript and interpreted the analysis. DZ conducted the literature review. WS reviewed the manuscript. BM and LM designed the study and reviewed the manuscript. All authors contributed to the article and approved the submitted version.

## Funding

This research was supported by the Second Affiliated Hospital of Guangzhou Medical University New Technology Clinical Research Project (2020-LCYJ-XJS-08).

## Conflict of interest

The authors declare that the research was conducted in the absence of any commercial or financial relationships that could be construed as a potential conflict of interest.

## Publisher’s note

All claims expressed in this article are solely those of the authors and do not necessarily represent those of their affiliated organizations, or those of the publisher, the editors and the reviewers. Any product that may be evaluated in this article, or claim that may be made by its manufacturer, is not guaranteed or endorsed by the publisher.
