# Supplementary material for: Relationship between human serum albumin and in-hospital mortality in critical care patients with chronic obstructive pulmonary disease
Source: Front Med (Lausanne). 2023 Apr 27;10:1109910. doi: 10.3389/fmed.2023.1109910 (PMC10174316; doi:10.3389/fmed.2023.1109910)
Supplement: Supplementary file 1 [file Data_Sheet_1.docx]

Stable1. Univariate Cox and multivariable Cox regression models evaluating the association between serum albumin and in-hospital mortality

| Variable | Unadjusted model  HR 95CI% | P value | Fully adjusted model  HR 95CI% | P value |
| --- | --- | --- | --- | --- |
| Serum albumin | 0.94 (0.93~0.96) | <0.001 | 0.97 (0.96~0.99) | 0.002 |
| Serum albumin groups |  |  |  |  |
| Albumin< 30g/L | 1(Ref) |  | 1(Ref) |  |
| Albumin≥ 30g/L | 0.52 (0.43~0.63) | <0.001 | 0.75 (0.60~0.93) | 0.009 |
| Age(years) | 1.04 (1.03~1.05) | <0.001 | 1.04 (1.03~1.05) | <0.001 |
| Sex, n (%)（male） | 1.08 (0.89~1.3) | 0.453 | 1.17 (0.95~1.43) | 0.133 |
| Glasgow score | 0.91 (0.89~0.93) | <0.001 | 1.03 (1.00~1.07) | 0.049 |
| MAP (mmHg) | 0.97 (0.96~0.98) | <0.001 | 1.00 (0.99~1.01) | 0.765 |
| Respiratory rate (BPM） | 1.09 (1.06~1.11) | <0.001 | 1.04 (1.01~1.07) | 0.005 |
| Oxygen saturation（%） | 0.88 (0.86~0.89) | <0.001 | 0.91 (0.89~0.93) | <0.001 |
| Serum sodium（mmol/L) | 1 (0.99~1.02) | 0.641 | 0.99 (0.97~1.00) | 0.097 |
| Serum potassium（mmol/L) | 1.26 (1.17~1.36) | <0.001 | 1.16 (1.06~1.28) | 0.002 |
| Creatinine (mg/L) | 1.05 (1.01~1.08) | 0.004 | 0.98 (0.91~1.04) | 0.494 |
| Hemoglobin (g/L) | 0.97 (0.93~1.02) | 0.233 | 1.00 (0.95~1.05) | 0.957 |
| Platelets（109/L) | 0.9989 (0.9979~0.9998) | 0.018 | 1.0008 (0.9998~1.0019) | 0.13 |
| PO2 | 0.9995 (0.9972~1.0017) | 0.635 | 1.0014 (0.9980~1.0048) | 0.421 |
| PCO2 | 1.01 (1.01~1.02) | <0.001 | 1.01 (1.01~1.02) | <0.001 |
| Myocardial infarction | 1.34 (1.08~1.65) | 0.007 | 1.04 (0.83~1.31) | 0.742 |
| Heart failure | 1.1 (0.91~1.33) | 0.346 | 0.82 (0.66~1.03) | 0.085 |
| Peripheral vascular disease | 0.89 (0.68~1.15) | 0.372 | 0.71 (0.54~0.94) | 0.017 |
| Cerebrovascular disease | 1.33 (1.03~1.71) | 0.028 | 1.27 (0.96~1.67) | 0.093 |
| Renal disease | 1.20 (0.99~1.46) | 0.07 | 0.75 (0.57~0.98) | 0.038 |
| Cancer | 1.41 (1.12~1.78) | 0.003 | 0.86 (0.64~1.14) | 0.293 |
| Diabetes mellitus | 0.78 (0.64~0.95) | 0.016 | 0.66 (0.53~0.83) | <0.001 |
| Sepsis | 2.08 (1.61~2.69) | <0.001 | 1.00 (0.74~1.35) | 0.999 |
| MV |  |  |  |  |
| Noninvasive-Ventilation | Ref. |  | Ref. |  |
| Invasive-Ventilation | 1.3 (0.91~1.87) | 0.149 | 0.75 (0.5~1.12) | 0.157 |
| No-Ventilation | 0.7 (0.49~1.01) | 0.055 | 0.81 (0.56~1.18) | 0.267 |
| Co-morbidity index | 1.15 (1.11~1.18) | <0.001 | 1.19 (1.13~1.26) | <0.001 |
| SAPSII score | 1.05 (1.05~1.06) | <0.001 | 1.01 (1.00~1.02) | 0.222 |
| SOFA score | 1.19 (1.16~1.21) | <0.001 | 1.16 (1.1~1.21) | <0.001 |
| OASIS score | 1.07 (1.06~1.08) | <0.001 | 1.01 (0.99~1.03) | 0.186 |
| COPD diagnoses sequence | 1.04 (1.02~1.05) | <0.001 | 1.00(0.99~1.02) | 0.75 |
| AECOPD | 1.00 (0.82~1.21) | 0.964 | 0.90 (0.73~1.1) | 0.304 |

Stable2. Multivariable Cox regression models evaluating the association between serum albumin and 30-day, 90-day and 1-year mortality.

| Exposure | 30-day mortality  HR(95% CI) | P value | 90-day mortality  HR(95% CI) | P value | 1-year mortality  HR(95% CI) | P value |
| --- | --- | --- | --- | --- | --- | --- |
| Serum albumin | 0.96 (0.94~0.98) | <0.001 | 0.96 (0.94~0.97) | <0.001 | 0.96 (0.95~0.98) | <0.001 |

A


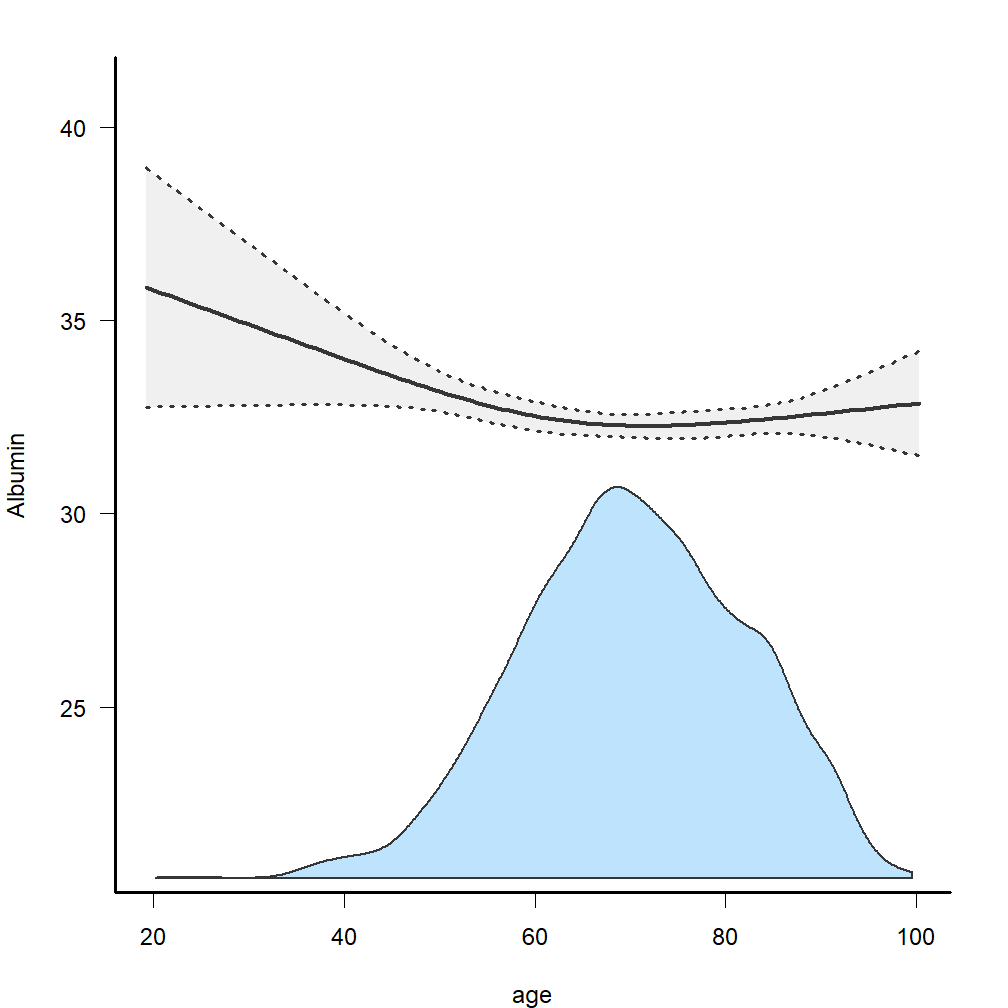


B


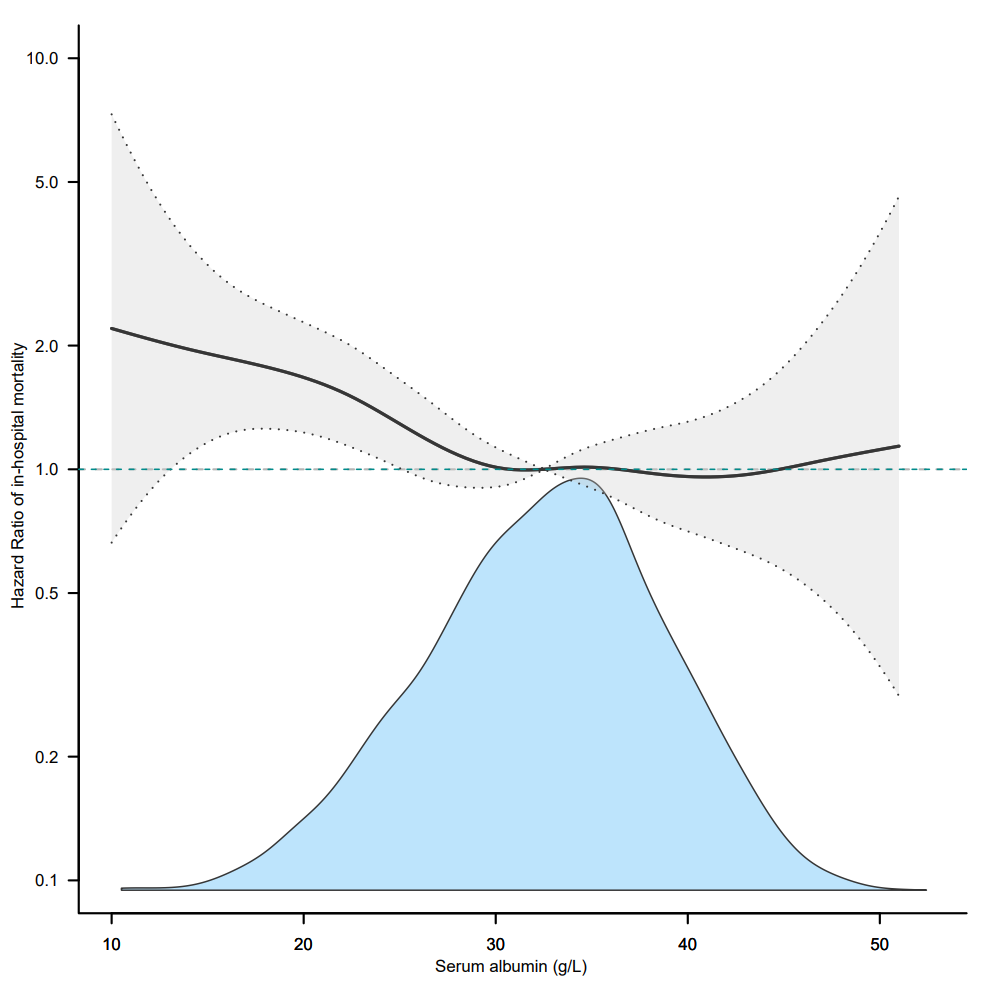


Sfigure 1: The relationship between age and albumin.

**
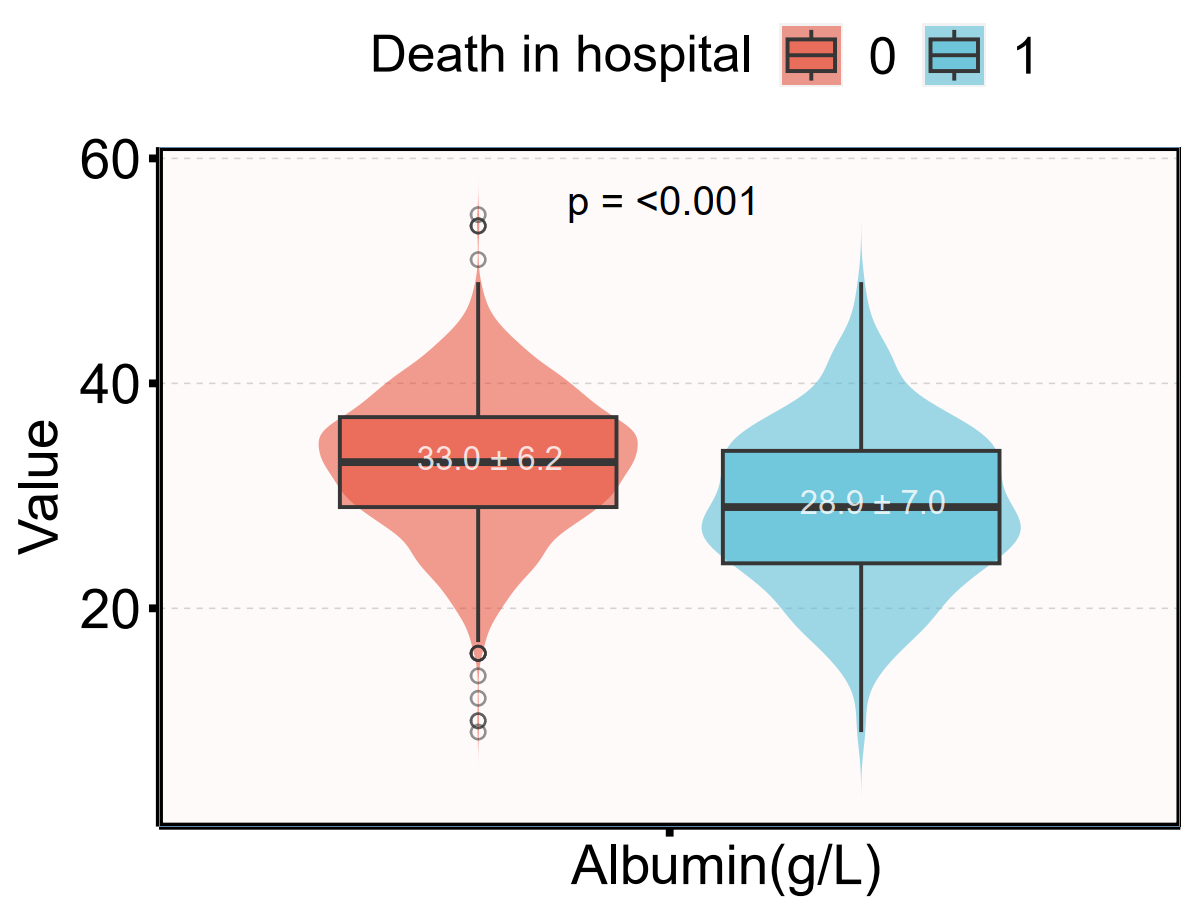
**

Sfigure 2: The distribution of albumin levels in those that survived versus those non-survivived.
